# The Frequency of Association of Nail Involvement and Psoriatic Arthritis in Psoriasis Patients

**DOI:** 10.5152/eurasianjmed.2023.53

**Published:** 2023-06-01

**Authors:** Erdal Pala, Mehmet Melikoğlu, Ömer Karaşahin, Meltem Alkan Melikoğlu

**Affiliations:** 1 Department of Dermatology and Venerology, Atatürk University, Faculty of Medicine, Erzurum, Turkey; 2 Erzurum Regional Training and Research Hospital, Infectious Diseases Erzurum, Turkey; 3 Department of Physical Therapy and Rehabilitation, Atatürk University, Faculty of Medicine, Erzurum, Turkey

**Keywords:** Psoriasis, psoriatic arthritis, nails, severity of illness index

## Abstract

**Objective:**

While the relationship between psoriatic arthritis and skin findings is well-known in patients with psoriasis, the relationship between psoriatic arthritis and nail involvement is less known. In this study, it was aimed to examine the frequency of association between nail involvement and psoriatic arthritis in patients with psoriasis.

**Materials and Methods:**

Our study is a retrospective observational study. It was conducted with 250 registered patients who applied to the dermatology polyclinic and clinic of our university hospital. The follow-up forms of the patients were scanned retrospectively and the findings were recorded.

**Results:**

The average age of the 250 patients evaluated in this study was 39.62 ± 9.30, and 133 (53.2%) of them were women. The frequency of nail involvement in psoriasis patients was determined to be 36.8% (n = 92) and the frequency of arthritis was determined to be 8.8% (n = 22). Nail involvement was statistically significantly more common in those with arthritis, and nail involvement was present in all of those with arthritis (*P* < .001). Nail involvement was significantly more common in those with only arthralgia (*P* < .001). A significantly higher average of nail psoriasis severity index was found in those with both joint and nail involvement compared to those with only nail involvement (*P* < .001). There was no statistically significant difference in terms of psoriasis area severity index average (*P* = .235). Proximal and distal interphalangeal arthralgia and sacroiliac arthralgia were found significantly more frequently in those with nail involvement than in those without nail involvement (respectively *P* = .007 and *P* < .001). There was no statistically significant relationship between nail involvement with the presence of arthritis and the clinical type (respectively *P* = .288 and *P* = .955).

**Conclusion:**

Joint involvement and nail involvement in patients with psoriasis are closely related, and we think that nail and joint involvement in psoriasis patients should be evaluated together.

**Main Points**

- Joint involvement and nail involvement in patients with psoriasis are closely related.
- Nail involvement was statistically significantly more common in those with arthritis.
- Concomitant arthritis in psoriasis may increase the nail psoriasis severity index score in patients.
- Distal interphalangeal joint complaints were more common in patients with nail findings in psoriasis.

## Introduction

Psoriasis is a systemic inflammatory, chronic and recurrent disease that might affect the skin, joints, and nails, with the frequency of 1%-3% in the population. Although immunological, autoimmune, and genetic factors are considered to be effective in the pathogenesis of the disease, it has not been fully clarified yet. Nail involvement is a common condition in patients with Ps. Although the normal frequency of nail involvement in psoriasis is 40%, this rate might increase up to 80% in cases with psoriatic arthritis (PsA).^1^ Psoriatic arthritis is a chronic immune-mediated spondyloarthropathy that may occur in 20-30% of patients with psoriasis and may lead to devastating and permanent joint damage. Peripheral arthritis, axial disease, dactylitis, enthesitis, and skin and nail psoriasis may also develop in PsA.^2^ Recently, it has been suggested that nail involvement in patients with psoriasis may be a marker of PsA development.^3^ Nail changes may occur 1-2 years before arthritis in patients with psoriasis.^4^ The relationship between nail involvement and distal interphalangeal (DIP) joint involvement is well known. It is believed that this close relationship occurs with the progression of the inflammation that starts in the nail and then progresses to the DIP joint due to the anatomical proximity of the nail bed and its matrix to this joint.^5^ Nail psoriasis is a condition that leads to a decrease in the self-confidence of individuals due to its disfiguring feature, along with an increase in the frequency of depression and anxiety. Likewise, PsA patients have deterioration in the quality of life, reduced functional capacity and loss of work force. Because of this, PsA results in negative consequences for the individual and society physically, psychologically, and economically. There is a need to establish a clinical indicator to detect the risk and early diagnosis of PsA.^3^ In the literature, studies on the frequency of association of PsA and nail involvement are very few. Therefore, in this study, we aimed to examine the frequency of nail involvement and PsA association in patients with psoriasis and how this association affects the nail psoriasis severity index (NAPSI) in patients. 

## Materials and Methods

The study is a retrospective cross-sectional study. Recorded patients, who were admitted to the Dermatology Polyclinic and Clinic between January 2018 and December 2022 and who were clinically and histopathologically diagnosed with psoriasis, were included in the study. The study was performed in accordance with the principles of the Declaration of Helsinki following receipt of approval from the local ethical committee (Date:December 29, 2022, No: 4). 

### Data Collection and Study Design

A total of 250 patients aged between 18 and 70 who were diagnosed with psoriasis clinically and histopathologically were included in the study. Pregnant and lactating women and people with another known inflammatory skin or systemic disease were excluded from the study. The demographic characteristics of the patients, duration of the disease, smoking and alcohol use, psoriasis clinical types, whether the patient has joint complaints, nail examination findings, medication use history, and body mass index (BMI) of the patients were retrospectively scanned and recorded in the registered files of the patients. Psoriasis area severity index (PASI) scores in patients were calculated and recorded according to the distribution areas of the lesions. According to the PASI results, those below 10 were considered mild, those between 10 and 15 were considered moderate, and those above 15 were considered severe diseases. Nail psoriasis severity index scores were calculated and recorded according to the nail examination findings and the number of nails involved. Whether those with joint complaints have swelling in the joints, joint stiffness when waking up in the morning, recurring pain in the tendons, pain and swelling in the fingers, how many joints and in which joint they have complaints were recorded according to the anamnesis files. In patients with joint complaints, the consultation notes requested from the Rheumatology Department of our hospital were examined using the electronic record system. According to the rheumatology note, patients who met the Classification Criteria for Psoriatic Arthritis (CASPAR) were considered to have arthritis. In the evaluation of the laboratory tests performed, the laboratory values of our hospital were taken into account for the reference values of the test results. In this context, as normal values; values of white blood cell (WBC) 4.000-11.000/µL, hemoglobin >12 g/dL, C-reactive protein (CRP) <5 mg/L, erythrocyte sedimentation rate 0-20 mm/h, rheumatoid factor (RF) 0-16 IU/mm were considered as normal values.

### Statistical Analysis

All tests were analyzed using the statistical package SPSS, version 21.0 (IBM Comp., Armonk, NY, USA). The Kolmogorov–Smirnov test was used to check the suitability of continuous variables to a normal distribution. In the comparison of 2 independent groups, the Mann–Whitney *U*-test was used for variables that do not have a normal distribution, and the Student’s *t*-test was used for variables that have a normal distribution. The relationships between categorical variables were tested by the chi-square test. It was accepted as *P* < .001 for statistical significance. 

## Results

### Demographic and Clinical Characteristics

The average age of the 250 patients evaluated in this study was 39.62 ± 9.30 (youngest–eldest; 20-67) and 133 (53.2%) were women. The demographic and clinical characteristics of the patients were presented in Table 1. The frequency of nail involvement in psoriasis patients was determined to be 36.8% (n = 92) and the frequency of arthritis was determined to be 8.8% (n = 22).

### Nail and Joint Involvement

A total of 67 of the patients with nail involvement (72.8%) had only fingernail involvement, 1 of them (0.4%) had only toenail involvement, and 24 of them (9.6%) had both fingernail and toenail involvement. The average of nail involvement number was 5.73 ± 2.23. Psoriatic hand and toenail findings in patients were presented in Table 2. Accordingly, the most common findings detected only on the fingernail and on both the toenail and the hand were pitting, onycholysis, and subungual hyperkeratosis while onycholysis, leukonychia, and subungual hyperkeratosis were detected on only the toenail of 1 patient. Pitting and onycholysis in 17 (18.5%) patients, pitting in 15 (16.3%) patients, and pitting, onycholysis, and subungual hyperkeratosis in 12 (13.0%) patients were detected.

**Figure 1.**
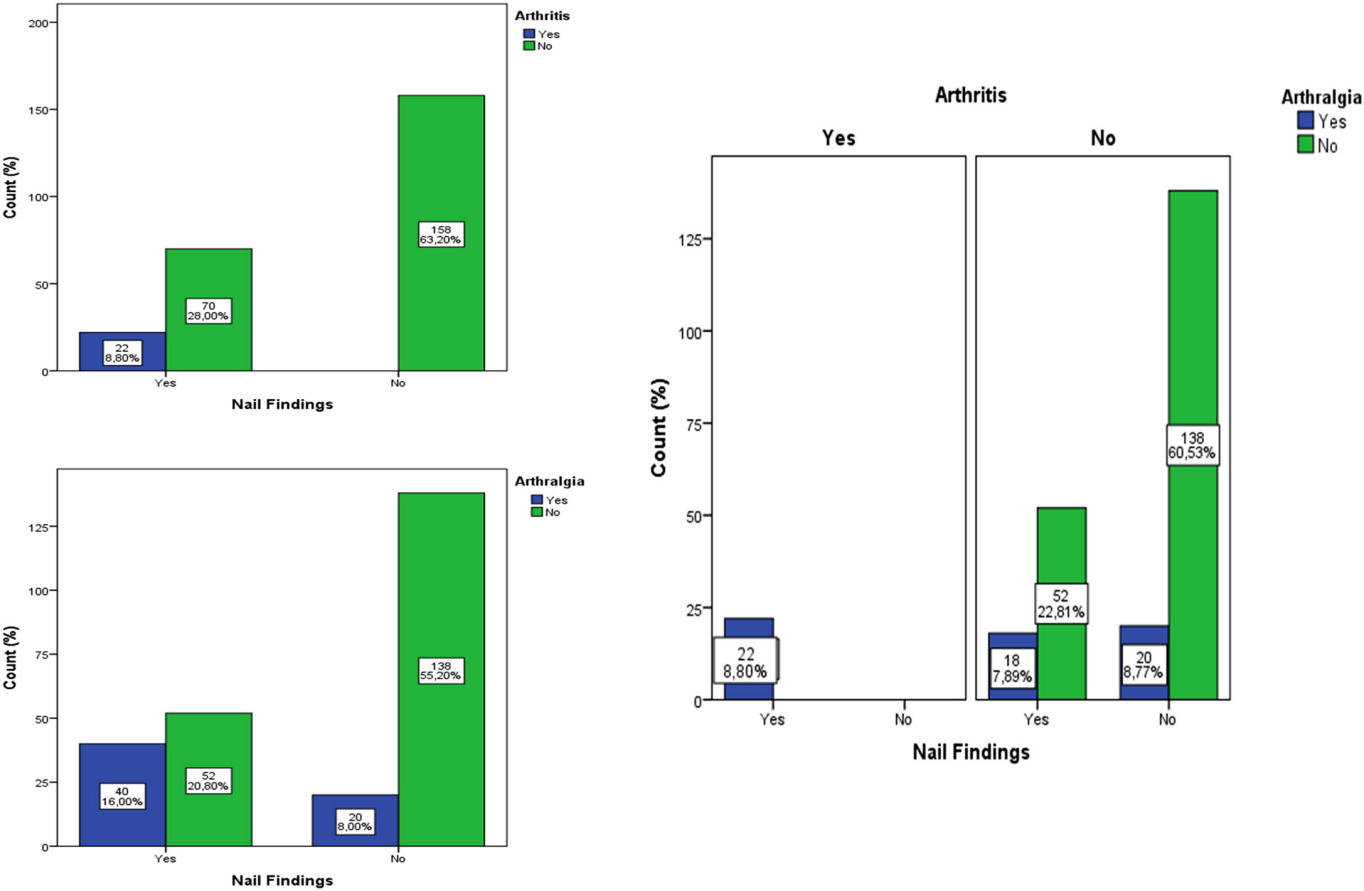
The relationship between nail involvement and arthritis and arthralgia.

The relationship between nail involvement, arthritis, and arthralgia is presented in Figure 1. Nail involvement was statistically significantly more common in those with arthritis, and nail involvement was present in all of those who developed arthritis (*P* < .001). Nail involvement was significantly more common in those with arthralgia (*P* < .001). Arthralgia and nail involvement were present in all 22 (8.8%) patients with arthritis. While 18 (7.89%) of 38 (16.6%) patients with arthralgia and no arthritis had nail involvement, 20 (8.77%) did not. A total of 138 (60.53%) patients did not have arthritis, arthralgia, or nail involvement.

**Figure 2.**
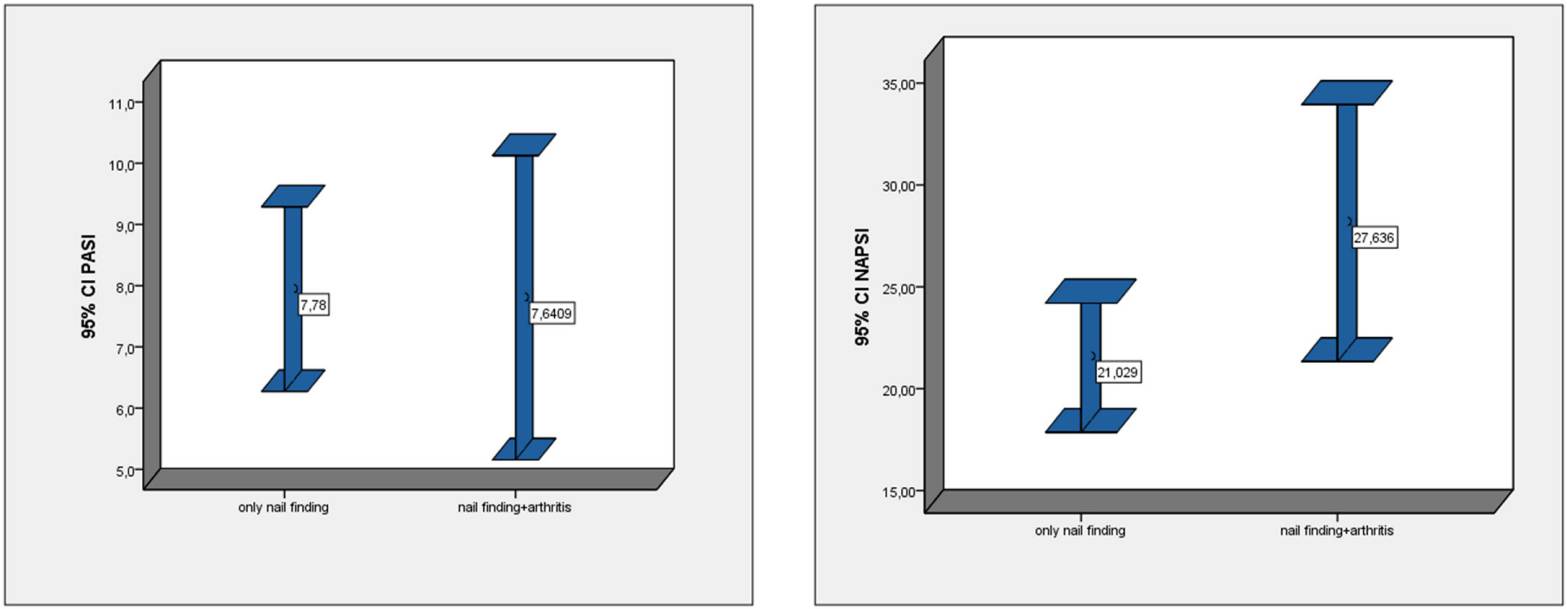
Average values of PASI and NAPSI according to nail and joint involvement.

Psoriasis area severity index and NAPSI values according to nail and joint involvement were presented in Figure 2. A significantly higher average of NAPSI was found in those with both joint and nail involvement compared to those with only nail involvement (*P* < .001). There was no statistically significant difference in terms of PASI average (*P* = .235).

The distribution of nail involvement according to the localization of joint pain is presented in Table 3. Proximal and DIP arthralgia and sacroiliac arthralgia were found significantly more frequently in those with nail involvement than in those without nail involvement (respectively *P* = .007 and *P* < .001).

The distribution of demographic, clinical, and laboratory characteristics according to the incidence of joint and nail involvement in psoriasis was evaluated in Table 4. The average RF was significantly higher in the presence of nail involvement (*P* < .001). Only one of the patients with arthritis was RF (4.5%) positive. Those with arthritis were found to be significantly older, with longer disease duration, and with higher BMI than those without arthritis (respectively *P* = .006, *P* = .004, and *P* = .009). Significantly higher CRP, sedimentation, and significantly lower hemoglobin were detected in those with arthritis (respectively *P* < .001 and *P* = .007).

**Figure 3.**
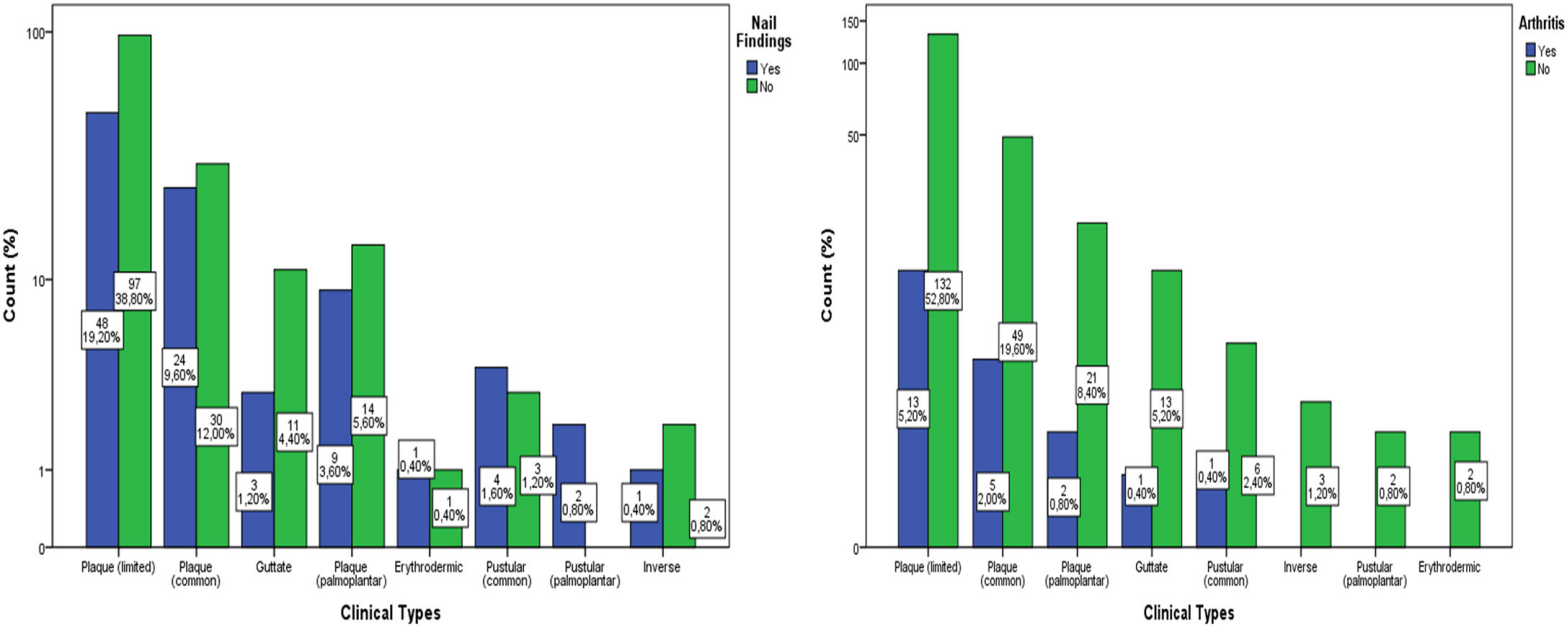
The distribution of clinical types according to the presence of nail and joint involvement in psoriasis.

The distribution of clinical types according to the presence of nail and joint involvement in psoriasis is shown in Figure 3. The most common clinical types of limited plaque, diffuse plaque, and palmoplantar plaque were detected in those with nail involvement. There was no statistically significant relationship between the presence of nail involvement and the clinical type (*P* = .288). Limited plaque, diffuse plaque, and palmoplantar plaque types were commonly observed in those with arthritis. There was no statistically significant relationship between the presence of arthritis and the clinical type (*P* = .955).

## Discussion

Psoriasis is a systemic inflammatory chronic and recurrent disease that affects the skin, nails, and joints and that might be accompanied by many comorbidities. Although the relationship between skin manifestations and joint findings in psoriasis is well known, there are fewer studies in the literature on the relationship between joint findings and nail findings. In psoriasis, both nail involvement and joint involvement impair the quality of life, working life, sleep quality, and general health status of patients, and create a great burden to both the individual and the society, physically, psychologically, and economically.^6,7^ There are opinions that the presence of nail involvement in patients with psoriasis may be a marker of PsA development. In this way, it is stated that the subclinical state of PsA might be detected early and its clinical progression might be prevented.^3^ Therefore, in this study, we aimed to examine the frequency of nail involvement and PsA association in patients with psoriasis and how this association affects the NAPSI in patients with PsA.

Psoriatic arthritis is a chronic, immune-mediated spondyloarthropathy that can be observed in patients with psoriasis. The incidence of PsA in patients with psoriasis varies between 7 and 40%.^8^ Symptoms such as inflammatory pain, diffuse arthralgia, arthritis, dactylitis, enthesitis, uveitis, peripheral edema, and nail involvement might be observed in the PsA clinic. Psoriatic arthritis might be seen clinically as polyarthritis, oligoarthritis, monoarthritis, DIP, spondylitis, and arthritis mutilans, involving the peripheral joint, spine, and enthesis region.^9^ In our patients, PsA was accompanied by a rate of 8.8% (n = 22) in 250 psoriasis patients in accordance with the literature. In our patients who were diagnosed with PsA, 50% (n = 11) monoarthritis, 9.1% (n = 2) oligoarthritis, 40.9% values were recorded. We can change the sentence like this. Despite this, although 24% (n = 60) of our patients have arthralgia complaints in one or several joints, it could not be decided whether PsA was present in these patients according to CASPAR criteria since the study was conducted retrospectively. In a study, it is stated that the average duration of arthralgia in patients with psoriasis is 35 months. Again in this study, it is stated that the long duration of psoriasis and the female gender increase the incidence of PsA.^10^ In our study, in accordance with the literature, it is found that the average duration of arthralgia was 33 months, the average duration of psoriasis was 12 years, and the frequency of female gender was higher in our patients. Those with arthritis were found to be significantly older, with longer disease duration, and with higher BMI than those without arthritis (respectively *P* = .006, *P* = .004, and *P* = .009). In our study, when the complaints of pain in the joint regions of the patients were evaluated, arthralgia was most common in the DIP and sacroiliac joints. Rheumatoid factor is very important in the separation of PsA and rheumatoid arthritis. Rheumatoid factor is positive in approximately 80% of patients with rheumatoid arthritis, while it is positive in 13% of patients with PSA.^4^ In this study, only 4.5% (n = 1) of 22 patients with arthritis were RF positive. 

Psoriasis is a disease that can also affect the nails. While nail involvement is mostly associated with skin manifestations, it might also be seen in the form of only nail involvement without skin manifestations in 5% of cases.^11^ There were no psoriasis patients with only nail findings in our study. All of our patients were accompanied with skin manifestations. Sometimes, only nail and joint involvement can be together. Nail involvement is observed in about 40% of patients with psoriasis, while this rate may be up to 80% if there is also joint involvement.^1^ In psoriasis, the fingernails are more often affected than the toenails. As there may be involvement in a single nail, there is often involvement in more than 1 nail.^12^ In our study, nail involvement was detected in 36.8% (n = 92) of the patients and, in accordance with the literature, the fingernails of the patients were most commonly involved. The average of nails involvement number in our patients was found 5.73 nails. Psoriatic nail findings can vary considerably between studies. In one study, the most common nail findings were reported as onycholysis and subungual hyperkeratosis.^13^ In our study, pitting and onycholysis were detected as the most frequent. This shows that both the nail matrix and the nail bed are often affected in our patients. 

The results of studies conducted on the frequency of nail involvement and PsA association in patients with psoriasis vary. It has been stated that the frequency of this association in some studies varies between 40% and 80%. In one study, it was reported that nail involvement was present in 64.4% of patients with DIP joint arthritis.^14^ In another study that nail involvement is examined according to PsA onset time, it is determined that the frequency of nail involvement in patients with early onset PSA was 77%, and the association of nail involvement in patients with late onset PSA was 74%.^15^ In this study, nail involvement was present in all of our patients with arthritis. However, nail involvement was again accompanied in 18 (7.89%) of the 38 (16.6%) patients who did not have arthritis, but had arthralgia and could not be diagnosed with PsA according to CASPAR criteria. Nail involvement was statistically significantly more common in those with arthritis, and nail involvement was present in all of those who developed arthritis. Nail involvement was significantly more common in those with arthralgia. Proximal and DIP arthralgia and sacroiliac arthralgia were found significantly more frequently in those with nail involvement than in those without nail involvement (respectively *P* = .007 and *P* < .001). C-reactive protein and sedimentation rate, which are indicators of inflammation, were statistically higher in patients with arthritis than in those without. 

The NAPSI is often used to determine the severity of nail involvement in patients with psoriasis. While pitting finding is scored according to the number of pitting in modified NAPSI (mNAPSI), other findings are scored according to the percentage of involvement. We used NAPSI scoring in our study. Palmou et al^16^ found no statistically significant difference in mNAPSI scores between psoriasis patients with and without PsA in a study. In another study, it was found that the mNAPSI score was higher in patients with DIP joint arthritis than in the control group.^17^ In our study, a statistically significant difference was found in terms of NAPSI between psoriasis patients with PsA and nail findings and patients with nail involvement without PsA. Nail involvement was more severe in those accompanied by PsA. Again, when the relationship between PsA and PASI in patients with psoriasis was examined in the literature, a significantly higher PASI score was observed in those with PsA.^18^ In this study, there was no statistically significant difference in terms of PASI score between patients with psoriasis with only nail findings and patients with nail involvement and joint involvement. There was no statistically significant relationship between clinical type in patients with only nail involvement and patients with nail involvement and joint involvement. The most common clinical types in patients with both only nail involvement and joint involvement along with nail involvement were determined as limited plaque, diffuse plaque, and palmoplantar plaque. 

### Limitations

There are some limitations to the study. First, the study was conducted retrospectively in a single center. Second, the fact that we could not compare it with the control group is another limitation of the study. Third, since we did not have access to the retrospective rheumatology consultation notes and rheumatology examination findings of all patients with arthralgia complaints in the patient registration forms, the decision on arthritis according to CASPAR criteria could not be made fully in these patients. This has created a limitation in terms of fully reflecting the frequency of arthritis in our patients. Fourth, the fact that we could not reach the records about the onset of nail findings in patients with and without arthritis constitutes a limitation. This constitutes a limitation in evaluating nail involvement as a predictor for PsA development. Therefore, we think that there is a need for prospective studies comparing a larger number of patients and control groups.

As a result, the frequency of nail involvement was found to be higher in patients with arthritis than in those without in this study. And again, it was found that the severity of nail involvement was higher in patients with joint involvement. While nail involvement can distinguish PsA from other causes of arthritis, it is also an important finding in predicting the development of PsA. Therefore, psoriasis patients, especially those who admit with nail involvement, should be closely monitored in terms of PsA development, and we think that rheumatologists should pay attention to nail examination with a multidisciplinary approach to such patients. 

## Figures and Tables

**Table 1.** Demographic and Clinical Characteristics of Patients

Variables		
Age, average ± SD		39.62 ± 9.30
Sex, n (%)	Male	117 (46.8)
	Female	133 (53.2)
Age of onset of the disease, average ± SD		27.42 ± 11.20
Duration of disease (month), average ± SD		143.04 ± 111.60
Body mass index, average ± SD		26.77 ± 4.22
Habits		
Presence of smoking, n (%)		96 (38.4)
Smoking (pack-year), average ± SD		14.13 ± 13.57
Presence of alcohol use, n (%)		4 (1.6)
Nail involvement, n (%)		92 (36.8)
	Hand	67 (72.8)
	Foot	1 (0.4)
	Hand–foot	24 (9.6)
Number of nail involvement, average ± SD		5.73 ± 2.23
Presence of arthritis, n (%)		22 (8.8)
	Monoarthritis	11 (50.0)
	Oligoarthritis	2 (9.1)
	Polyarthritis	9 (40.9)
Presence of arthralgia, n (%)		60 (24.0)
	Active	18 (30.0)
	Inactive	42 (70.0)
Duration of arthralgia (month), average ± SD		33.08 ± 28.26
NAPSI score, average ± SD		8.32 ± 13.72
PASI score, average ± SD		7.71 ± 5.87

NAPSI, nail psoriasis severity index; PASI, psoriasis area severity index.

**Table 2.** Psoriatic Hand and Toe Nail Findings in Patients

Findings, n (%)	Hand Nail (n = 67)	Foot Toe (n = 1)	Hand–Toe Nail (n = 24)
Pitting	58 (86.6)	–	21 (87.5)
Onycholysis	45 (67.2)	1 (100)	21 (87.5)
Subungual hyperkeratosis	25 (37.3)	1 (100)	14 (58.3)
Leukonychia	8 (11.9)	1 (100)	6 (25.0)
Breaking quickly	10 (14.9)	–	1 (4.2)
Splinter hemorrhage	6 (9.0)	–	8 (33.3)
Red lunula	–	–	–

**Table 3.** Distribution of Nail Involvement According to Localization of Joint Pain

	Nail Finding		*P*
	Yes (n = 92)	No (n = 158)	
Cervical arthralgia	–	2 (1.3%)	.398
Shoulder arthralgia	–	1 (0.6%)	.632
Elbow arthralgia	1 (1.1%)	2 (1.3%)	.694
Wrist arthralgia	8 (8.7%)	6 (3.8%)	.102
Proximal interphalangeal arthralgia	9 (9.8%)	3 (1.9%)	**.007**
Distal interphalangeal arthralgia	20 (21.7%)	5 (3.2%)	**<.001**
Thoracic arthralgia	–	–	–
Lumbar arthralgia	7 (7.6%)	5 (3.2%)	.113
Sacroiliac arthralgia	20 (21.7%)	8 (5.1%)	**<.001**
Knee arthralgia	4 (4.3%)	1 (0.6%)	.063
Ankle arthralgia	3 (3.3%)	–	.050

**Table 4.** Distribution of Demographic, Clinical, and Laboratory Characteristics According to the Incidence of Joint and Nail Involvement in Psoriasis

	Nail		*P*	Arthritis		*P*
	Yes	No		Yes	No	
Age, average ± SD	40.57 ± 9.15	39.08 ± 9.37	.231	45.05 ± 7.97	39.10 ± 9.27	**.006**
Sex, n (%) female	42 (31.6%)	91 (68.4%)	.068	15 (68.2%)	118 (51.8%)	.140
Male	50 (42.7%)	67 (57.3%)		7 (31.8%)	110 (48.2%)	
Age of onset of the disease, average ± SD	27.01 ± 11.06	27.67 ± 11.32	.657	27.13 ± 9.15	27.45 ± 11.40	.984
Duration of disease (month), average ± SD	169 ± 127	134 ± 105	.180	210 ± 133	137 ± 107	**.004**
Body mass index, average ± SD	26.68 ± 3.90	26.82 ± 4.41	.980	29.10 ± 4.37	26.54 ± 4.15	**.009**
Habits						
Presence of smoking, n (%)	42 (43.8%)	54 (56.3%)	.072	8 (8.3%)	88 (91.7%)	.837
Smoking (pack-year), average ± SD	12.62 ± 8.61	14.54 ± 14.66	.392	7.91 ± 3.53	14.76 ± 14.06	.183
Presence of alcohol use, n (%)	2 (50.0%)	2 (50.0%)	.469	–	4 (100%)	.690
Laboratory, average ± SD						
C-reactive protein	6.89 ± 8.06	4.65 ± 4.65	.111	10.55 ± 7.76	4.99 ± 5.83	**<.001**
Sedimentation	13.28 ± 14.9	10.34 ± 10.27	.901	30.86 ± 19.60	9.55 ± 9.40	**<.001**
Rheumatoid factor	5.86 ± 3.05	4.27 ± 2.20	**<.001**	7.95 ± 4.01	4.56 ± 2.29	**<.001**
White blood cell	8.06 ± 2.11	8.14 ± 2.26	.965	8.23 ± 2.64	8.10 ± 2.29	.785
Hemoglobin	14.60 ± 2.04	14.54 ± 1.71	.587	13.47 ± 1.91	14.67 ± 1.79	**.007**
Thrombocyte count	289 ± 80	280 ± 66	.478	319 ± 106	278 ± 65	.065
